# Distinct roles of synaptic and extrasynaptic GABA_A_receptors in striatal inhibition dynamics

**DOI:** 10.3389/fncir.2013.00186

**Published:** 2013-11-26

**Authors:** Ruixi Luo, John G. Partridge, Stefano Vicini

**Affiliations:** Department of Pharmacology and Physiology, Georgetown University School of MedicineWashington, DC, USA

**Keywords:** GABA, tonic inhibition, striatum, patch-clamp, Cre-lox genetics

## Abstract

Striatonigral and striatopallidal projecting medium spiny neurons (MSNs) express dopamine D1 (D1+) and D2 receptors (D2+), respectively. Both classes receive extensive GABAergic input via expression of synaptic, perisynaptic, and extrasynaptic GABA_A_ receptors. The activation patterns of different presynaptic GABAergic neurons produce transient and sustained GABA_A_ receptor-mediated conductance that fulfill distinct physiological roles. We performed single and dual whole cell recordings from striatal neurons in mice expressing fluorescent proteins in interneurons and MSNs. We report specific inhibitory dynamics produced by distinct activation patterns of presynaptic GABAergic neurons as source of synaptic, perisynaptic, and extrasynaptic inhibition. Synaptic GABA_A_ receptors in MSNs contain the α2, γ2, and a β subunit. In addition, there is evidence for the developmental increase of the α1 subunit that contributes to faster inhibitory post-synaptic current (IPSC). Tonic GABAergic currents in MSNs from adult mice are carried by extrasynaptic receptors containing the α4 and δ subunit, while in younger mice this current is mediated by receptors that contain the α5 subunit. Both forms of tonic currents are differentially expressed in D1+ and D2+ MSNs. This study extends these findings by relating presynaptic activation with pharmacological analysis of inhibitory conductance in mice where the β3 subunit is conditionally removed in fluorescently labeled D2+ MSNs and in mice with global deletion of the δ subunit. Our results show that responses to low doses of gaboxadol (2 μM), a GABA_A _receptor agonist with preference to δ subunit, are abolished in the δ but not the β3 subunit knock out mice. This suggests that the β3 subunit is not a component of the adult extrasynaptic receptor pool, in contrast to what has been shown for tonic current in young mice. Deletion of the β3 subunit from D2+ MSNs however, removed slow spontaneous IPSCs, implicating its role in mediating synaptic input from striatal neurogliaform interneurons.

## INTRODUCTION

Striatonigral medium spiny neurons (MSNs) express dopamine D1 receptors (D1+) while striatopallidal MSNs express dopamine D2 receptors (D2+). Recent evidence (Cui et al., 2013) questions the traditional view that D2+ MSNs inhibit while D1+ MSNs facilitate movement initiation (Kravitz et al., 2010). GABAergic regulation of the intrinsic excitability of MSN is crucial to their role in striatal function (Ade et al., 2008; Paille et al., 2013).

GABAergic inhibition is mediated by a combination of fast, phasic inhibition and slow, tonic inhibition (Farrant and Nusser, 2005; Glykys and Mody, 2007). Synaptically released GABA mediates phasic inhibition through low-affinity GABA_A_ receptors and is effective in generating neuronal rhythmic activity and synchronicity (Cobb et al., 1995). GABA_A _receptor mediated tonic current can be both agonist-dependent and independent (Wlodarczyk et al., 2013). GABA spillover from vesicular and non-vesicular sources activates high-affinity extrasynaptic receptors to increase input conductance, affecting neuronal excitability (Brickley et al., 1996; Walker and Semyanov, 2008). In addition perisynaptic GABA receptor mediates an intermediate form of inhibition that is mediated by the neurogliaform subtype of neuropeptide Y positive interneurons (NGF-NPY; Capogna and Pearce, 2011; English et al., 2011). The subunit composition of GABA_A_ receptors that mediate striatal tonic inhibition are of crucial interest in understanding the underlying factors that control MSN output and neuronal excitability. Our previous study used pharmacology (Janssen et al., 2009) and Cre-lox genetics (Janssen et al., 2011) to identify the β3 subunit of GABA_A_ receptor as an important regulator of both striatal D1+ and D2+ MSN tonic current. Here we further extend those findings relating presynaptic activation with pharmacological analysis of inhibitory conductance in mice where the β3 subunit is conditionally removed in fluorescently labeled D2+ MSNs and in mice with global deletion of the δ subunit. We also investigate the role of two classes of presynaptic GABAergic interneurons (Gittis and Kreitzer, 2012), the fast spiking parvalbumin expressing (FS-PV) and NGF-NPY, in distinct GABAergic conductances.

## MATERIALS AND METHODS

### ANIMALS

The use of several strains of transgenic mice examined in this study to genetically identify rare striatal interneurons and have been described previously (Luo et al., 2013).These include the BAC-*npy-*eGFP and *parv*-Cre; *rosa26*-tdTom. Conditional and neuron specific GABA_A_ β3 subunit knockout (KO) mice and appropriate control mice were produced as reported in Janssen et al., 2011, by crossing floxed β3 mice (β3^f/f^) (Jackson Labs # 008310; Ferguson et al., 2007) to transgenic mice that express Cre recombinase under the *drd2* promoter (GENSAT, ER44; Gong et al., 2007). These Cre-dependent β3 subunit KO mice were subsequently crossed to “floxed-stop” *rosa26*-tdTom mice (Jackson Labs # 007914; Madisen et al., 2010); a second Cre-dependent line to allow visual identification of neurons lacking the β3 receptor subunit restricted to D2+ neurons. We designate this line as *drd2*-Cre;β3^f/f^;Rosa^tdTom^. Control mice used in these studies included *drd2*-Cre;β3^f/^^+^;Rosa^tdTom^ and Cre-negative β3^f/f^;Rosa^tdTom^. We did not detect any statistical differences between these two genotypes and combined them for control experiments. We employed commercially available genotyping of tail biopsies of littermates via Transnetyx, Inc. (Cordova, TN, USA) before conducting experiments. We also used global GABA_A_ δ-receptor subunit knockout mice previously described (Mihalek et al., 1999; Janssen et al., 2009).

### BRAIN SLICE PREPARATION

Male and female mice (post-natal day 14–18) were sacrificed by decapitation in agreement with the guidelines of the AMVA Panel on Euthanasia and the Georgetown University ACUC. The whole brain was removed and placed in an ice-cold slicing solution containing (in mM): NaCl (85), KCl (2.5), CaCl_2_ (1), MgCl_2_ (4), NaH_2_PO_4_ (1), NaHCO_3_ (25), glucose (25), sucrose (75) (all from Sigma, St. Louis, MO, USA). Coronal slices (250 μm) containing striatum were prepared using a Vibratome 3000 Plus Sectioning System (Vibratome, St. Louis, MO, USA) in slicing solution. Upon hemi-sectioning, tissue was incubated in artificial cerebrospinal fluid (aCSF) containing (in mM): NaCl (124), KCl (4.5), Na_2_HPO_4_ (1.2), NaHCO_3_ (26), CaCl2 (2.0), MgCl2 (1), and dextrose (10.0) at 305 mOsm at 32°C for 30 min. The slices were incubated for an additional 30 min in aCSF at room temperature. All solutions were maintained at pH 7.4 by continuous bubbling with 95% O_2_/5% CO_2_.

### WHOLE-CELL RECORDINGS

Acute slices were visualized under an upright microscope (E600FN, Nikon) equipped with Nomarski optics and a 60× water immersion objective with a long working distance (2 mm) and high numerical aperture (1.0). Neurons were identified with green or red fluorescent protein expression, based on animal genotype. Identification of fluorescent protein-expressing neurons was performed by epi-fluorescent excitation of the tissue with a mercury-based lamp and standard filter sets. Recording pipettes, 4–6 MΩ in resistance, were prepared from borosilicate glass capillaries (Wiretrol II; Drummond) and filled with potassium chloride (KCl) or potassium gluconate (Kgluc)-based (for interneurons) internal solutions. The KCl-based internal solution contained (in mM): KCl, 145; HEPES, 10; ATP•Mg, 5; GTP•;Na, 0.2; EGTA, 5; adjusted to pH 7.2 with KOH. This high chloride internal solution enhanced the detection of GABAergic events, placing their reversal potential near 0 mV. In Kgluc-based internal solutions, KCl was replaced with equimolar (145 mM) Kgluconate and pH was adjusted with KOH.

All recordings presented here were performed at room temperature, 22–24°C. Voltage-clamp recordings were achieved using the whole-cell configuration of the patch-clamp technique at a holding potential of -70 mV using the Multiclamp 700B amplifier (Molecular Device Co., Sunnyvale, CA, USA). Synaptically coupled interneurons were subjected to holding current values which hyperpolarized neurons to a resting membrane potential of ~-70 mV in current-clamp mode. Access resistance was monitored periodically during recordings and experiments with >20% change were discarded. Membrane potential measurements were not corrected for liquid junction potential errors.

Stock solutions of bicuculline methobromide (BMR), tetrodotoxin (TTX), 4,5,6,7-tetrahydroisoxazolo(5,4-c)pyridine-3-ol, (THIP, gaboxadol), and sodium-2,3-dihydro-6-nitro-7-sulfamoyl-benzo[f]quinoxaline (NBQX) (all from Abcam Biochemicals Cambridge, MA, USA) were prepared in water. Etomidate (Sigma) was dissolved in dimethylsulfoxide at a 30 mM stock solution (the working solution of 0.5 μM etomidate corresponds to a final concentration of 0.002% DMSO). Stock solutions were diluted to the desired working concentration in aCSF and applied locally through a Y tube (Murase et al., 1989; Hevers and Lüddens, 2002).

Recordings were filtered at 2 kHz with a low-pass Bessel filter and digitized at 5–10 kHz using a personal computer equipped with Digidata 1322A data acquisition board and pCLAMP10 software (both from Molecular Devices). Data analysis, curve fitting, and Figure preparation were performed with Clampfit10 software (Molecular Devices). Spontaneous or miniature inhibitory post-synaptic currents (sIPSCs and mIPSCs) were identified using a semi-automated threshold-based mini detection software (Mini Analysis, Synaptosoft Inc., Fort Lee, NJ, USA) and were visually confirmed. IPSC averages were based on more than 50 non-overlapping events, and decay kinetics were determined with averaged IPSC traces using double exponential curve fittings and reported as weighted time constants (*T*_w_):

$$T_{w}=t_{1}*\left[A_{1}/\left(A_{1}+A_{2}\right)\right]+t_{2}*\left[A_{2}/\left(A_{1}+A_{2}\right)\right]$$

where *t*_n_ is the decay time constant for a particular component of the curve and *A*_x_ is the peak amplitude of the corresponding component. Similarly, decay time constants of unitary evoked IPSCs in synaptically coupled pairs were determined from the average of N evoked responses. mIPSCs were isolated with TTX (0.5 μM) and NBQX (5 μM) while NBQX was not included in sIPSC measurements to not perturb the network activity after removing rapidly decaying sEPSCs as previously described (Forcelli et al., 2012).

THIP mediated inward currents were primarily measured with an all-points histogram that measured the mean holding current 10 s before and during drug application (Luo et al., 2013). Holding current changes during high frequency stimulation of synaptically connected pairs were assessed during depolarization of presynaptic neurons and low pass filtering of the post-synaptic current recording (2–5 Hz).

### STATISTICS

Box and whisker plots were generated for more accurate representation of critical data. The whiskers include the minimum and maximum values, while the box outlines the 25th and 75th percentile of data points. The median value is represented by a bar inside the box. Other summary plots display the mean and error bars denote the SEM for a given data set.

Statistical significance was determined using the paired two-tailed Student’s *t*-test to compare pre-drug conditions with recordings made under drug conditions (etomidate) of the same cell population. Kolmogorov–Smirnov tests were conducted on all data sets to check for normal distribution and appropriate statistics were chosen dependent upon these results. Significance criteria were set at *p* ≤ 0.05, and all values in text and figures are expressed as mean ± SEM.

## RESULTS

**FIGURE 1:**
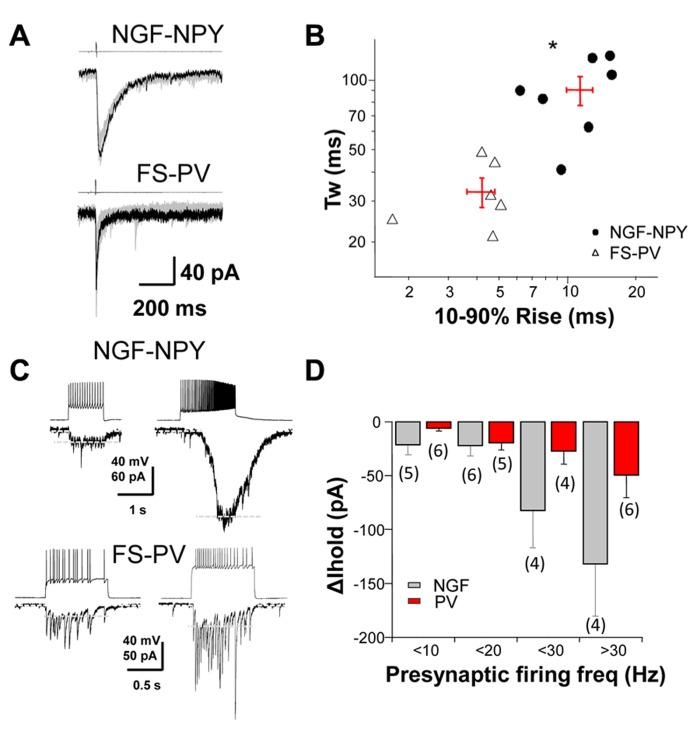
** GABAergic dynamics at interneuron-MSN synapses. (A)** A depolarization step to +40 mV (4 ms in duration) in a NGF-NPY and FS-PV interneuron induced slow and fast IPSCs, respectively, in synaptically connected MSNs. **(B)** scatterplot in log-log scale of the 10–90% rise time versus the weighted decay time for IPSCs in MSNs in response to action potentials in NGF-NPY and FS-PV interneuron. Each point represents one MSN, with average shown as + in red. IPSCs by FS-PV have faster rise and decay kinetics than NGF-NPY interneurons. ^*^p < 0.05 unpaired two-tailed Student’s *t* test. **(C)** Depolarization steps elicit action potentials in NGF-NPY and FS-PV interneurons that evoke GABAergic current in synaptically connected MSNs. High frequency action potential firing in NGF-NPY interneuron induced a large shift in holding current, while similar frequency in FS-PV interneuron induced only a moderate shift. Holding current (∆ *I*_hold_) as indicated by white dashed lines was assessed after low pass filtering (2–5 Hz). Note that individual IPSCs in the FS-PV paired MSN persist under high frequency. **(D)** Summary of the average shift in holding current for presynaptic firing frequencies of <10, <20, <30, and >30 Hz.

To investigate the role of synaptic, perisynaptic and extrasynaptic GABA_A_ receptors in striatal neurons we performed dual whole cell recordings from MSNs and two subtypes of GABAergic interneurons, the NGF-NPY and FS-PV, in mice expressing fluorescent markers (Luo et al., 2013). Single presynaptic action potentials produced reliable IPSCs when neurons were synaptically coupled (**Figure 1A)**. The rise and decay time of evoked IPSCs from these two interneuron classes differed significantly (**Figure 1B**). We then characterized the change in holding current (Δ *I*_hold_) in post-synaptic MSNs in response to increasing action potential frequency of presynaptic interneurons (**Figure 1C**). **Figure 1D** summarizes these data and illustrates the occurrence of tonic current in MSNs resulting from presynaptic activation.

Tonic GABAergic currents in MSNs from adult mice are carried by extrasynaptic receptors containing the α4 and δ subunit (Santhakumar et al., 2010), while in younger mice this current is mediated by receptors that contain the α5 and β3 subunit (Ade et al., 2008; Janssen et al., 2009). These studies have shown that deletion of the δ subunit removes GABAergic tonic current from adult MSNs while deletion of the β3 subunit removes tonic current from young MSNs. Although α4 and δ subunit mediated tonic current has been found in younger MSNs, it does not differ between D1+ and D2+ subtypes (Janssen et al., 2009). It is the differential expression of the β3 subunit that has been suggested to account for the observed difference in tonic current between D1+ and D2+ subtypes.

**FIGURE 2:**
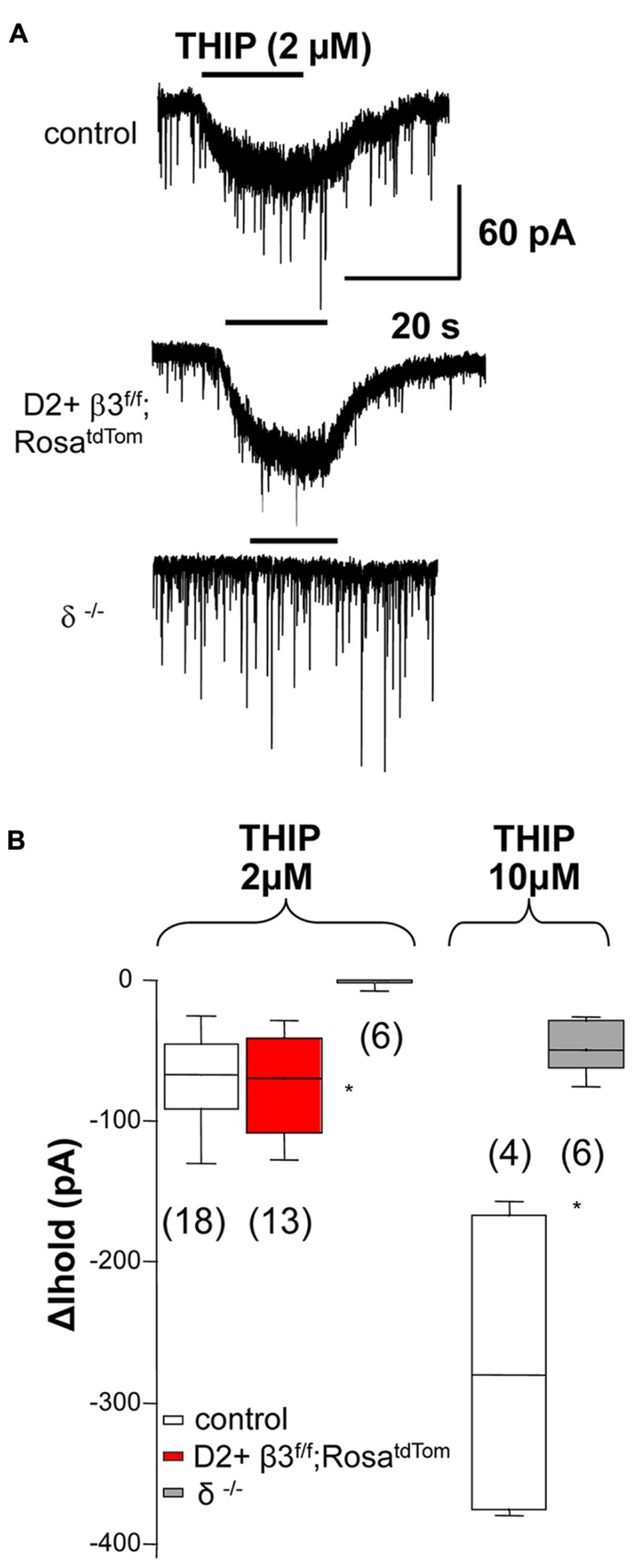
** The β3 subunit does not associate with the δ subunit in extrasynaptic GABA _A_ receptors. (A)** Example traces illustrating 2 μM THIP induced current in control MSNs and D2+ MSNs from β3^f/f^;Rosa^tdTom^ mice and the lack of response in MSN from δ^-^^/^^-^ mice Summary of the data obtained is shown in **(B)** together with the summary of currents (Δ*I*_hold_) elicited by 10 μM THIP compared between MSN from control and δ^-^^/^^-^ mice. Note that at 10 μM THIP begins to activate non δ-containing receptors ^*^p < 0.05. One-way ANOVA for repeated measures followed by Dunn’s *post hoc* test for the 2 μM THIP cell groups while the Mann–Whitney test was used for the 10 μM THIP groups.

To investigate if deletion of the β3 subunit also affects tonic currents mediated by α4 and δ subunit, we used THIP (gaboxadol), a δ subunit-containing GABA_A_ receptor superagonist (Brown et al., 2002). As shown in examples in **Figure 2A** and the summary data in **Figure 2B**, response to low doses of gaboxadol (2 μM) are abolished in δ but not β3 subunit knock out mice. This suggests that, in contrast to what was previously shown for tonic current in young mice (Janssen et al., 2009), the β3 subunit is not a component of δ subunit-containing extrasynaptic receptor pool in adult mice (Santhakumar et al., 2010).

**FIGURE 3:**
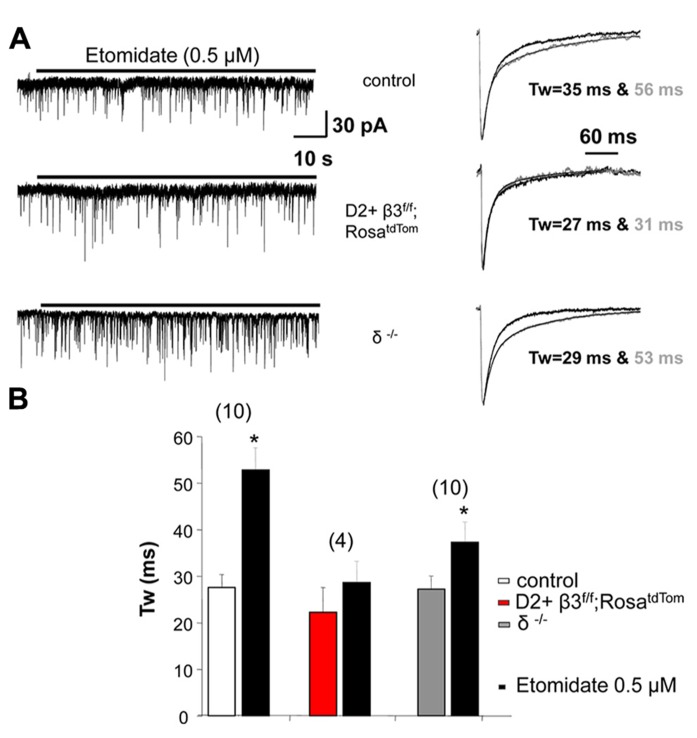
**GABA _A_ receptor subunits affect response to etomidate. (A)** Example traces at a slow time scale (*left*) illustrating the lack of effect of 0.5 μM etomidate on holding current in D2+ MSNs deriving from control and β3^f/f^;Rosa^tdTom^ compared to unidentified MSNs from δ^-^^/^^-^ mice. On the right are shown superimposed the average normalized sIPSCs with exponential fits and weighted decay time constant in the absence and the presence of etomidate. **(B)** Summary graph comparing the weighted decay time constant of average sIPSCs measured in MSNs from the three genotypes in the absence and the presence of 0.5 μM etomidate. ^*^p < 0.05 paired two-tailed Student’s *t* test (control group) or Wilcoxon matched pairs test. The average increase in *T*_w_ in δ^-^^/^^-^ MSNs is less compared to control but the difference is not significant.

Removing the β3 subunit of GABA_A_ receptors from MSNs reduced whole cell current mediated by the general anesthetic etomidate, a β3 subunit preferring modulator (Janssen et al., 2011). Here we extend these results by investigating the action of this general anesthetic on sIPSCs recorded from fluorescently identified D2+ MSNs where the β3 subunit is conditionally deleted. We used a concentration of etomidate (0.5 μM) that does not produce a sustained tonic current (**Figure 3A)**. As shown in the example traces in **Figure 3A** and the summary data in **Figure 3B**, etomidate (0.5 μM) doubled the decay time of sIPSCs in control D2+ MSNs but not in D2+ MSNs with the β3 subunit removed. sIPSCs from MSNs in δ subunit ^-^^/^^-^ mice were also significantly prolonged by etomidate.

**FIGURE 4:**
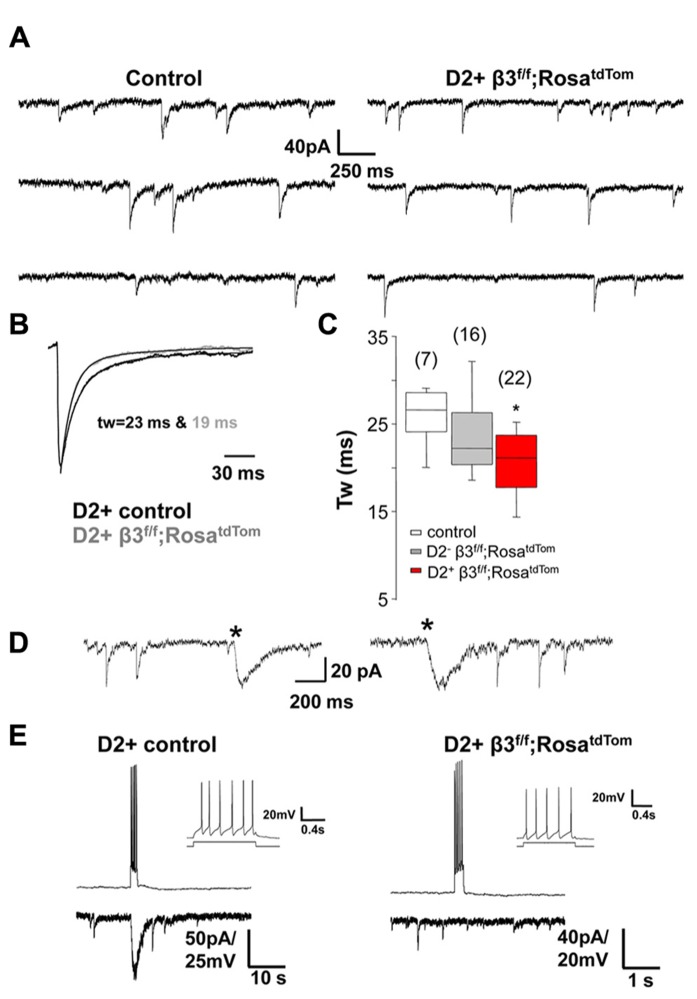
** Synaptic decay is altered specifically in β3^-/-^ MSNs. (A)** Representative raw traces of mIPSCs from a control D2+ MSN and a β3^f/f^;Rosa^tdTom^ D2+ MSNs. **(B)** Average normalized mIPSCs with double exponential fitting illustrating difference in *T*_w_ between the two cells shown in A. **(C)** Box plot summarizing the weighted decay time constant of average mIPSCs measured in MSNs from the three genotypes as indicated ^*^p < 0.05. One-way ANOVA for repeated measures followed by Tukey’s *post hoc* test. **(D)** Representative traces of the rarely occurring spontaneous slow IPSCs (^*^) from two MSNs in different control mice. Slow IPSC were identified by the distinctively slow rise and decay kinetics. **(E)** Burst of action potentials in a presynaptic NGF-NPY neuron elicited a large slow IPSC only in the D2+ control neuron (left) and not in the β3^-^^/^^-^ D2+ MSNs. The NGF-NPY cells were identified in the β3^-^^/^^-^ mice by soma size and the characteristic firing pattern (inset).

Our previous results revealed that mIPSCs in unidentified MSNs from mice where the β3 subunit was conditionally deleted in D2+ MSNs were significantly faster than in control littermates (Janssen et al., 2011). We refined these results by recording from fluorescent D2+ MSNs and non fluorescence (D2-) MSNs in newly developed mice where the β3 subunit is conditionally deleted in D2+ MSNs that also express red fluorescence* (drd2*-Cre;β3 ^f/f^;Rosa^tdTom^). Individual mIPSCs recorded in voltage clamp from control and β3 subunit KO MSNs (**Figure 4A**) are averaged and fitted with double exponential decay to show the difference in decay kinetics (**Figure 4B**). As seen in the summary results (**Figure 4C**), in D2+ MSNs with the β3 subunit removed, mIPSCs decay faster compared to MSNs in control mice and also D2- MSNs in KO mice. Hentschke et al. (2009) showed that deletion of the β3 subunit in hippocampal CA1 pyramidal neurons removes slow sIPSCs. Therefore we speculate that another contributing factor to the change in average mIPSC kinetics in D2+ MSNs with deletion of the β3 subunit could be the lack of slow IPSCs mediated by presynaptic NGF-NPY interneurons, as shown in the examples in **Figure 4D**. To investigate this, we looked for the occurrence of slow sIPSCs in several D2+ MSNs in control and in mice with β3 subunit conditional deletion. Spontaneous slow IPSCs occurred very infrequently (<1 event/5 min), in 12 % (6/51) of D2+ MSNs from 10 control mice, 13 % (4/31) of MSNs from 5 δ subunit -/- mice, but they were not observed in 58 D2+ MSNs from 13 mice with the β3 subunit deletion. The differences observed were statistically significant for D2+ MSNs in mice with β3 subunit conditional deletion compared to wild type or δ subunit -/- mice (*P* = 0.009 and 0.013; Fisher’s Exact Test). This is supported by pair recordings from putative NGF-NPY interneurons and D2+ MSNs with (**Figure 4E** left) and without the β3 subunit (**Figure 4E** right). The NGF-NPY cells were identified in the β3 subunit conditional deletion mice by soma size and characteristic firing pattern (**Figure 4E** inset, Ibáñez-Sandoval et al., 2011 and Luo et al., 2013). As illustrated a burst of action potentials in the NGF-NPY neurons elicited a large slow IPSC only in the D2+ control neuron and not in β3 subunit lacking D2+ MSN. A lack of slow synaptic responses were observed in four additional NGF-NPY interneuron and D2+ MSN pairs in β3 subunit conditional deletion mice. To further investigate this we recorded from D2+ MSNs during local application of extracellular solution with 12 mM K^+^ or 50 μM 4-aminopyridine to increase synaptic activity. Under these conditions we observed occasional bursts of action potentials in NGF-NPY neurons and large slow IPSCs in D2+ MSNs. In addition, slow IPSCs occurred in 60 % (9/15) of D2+ MSNs from 3 control mice, 75 % (3/4) of MSNs from 1 δ subunit -/- mouse, but they were observed only in 1 out of 10 D2+ MSNs from 2 mice with the β3 subunit deletion (*P* = 0.018 and 0.041; Fisher’s Exact Test). Taken together these data suggest that the role of β3 subunits in mediating synaptic input from neurogliaform neurons as seen in the hippocampus is likely to hold for the striatum as well. But given the rare occurrence of slow IPSCs, their presence would not cause significant changes in the average mIPSC kinetics. Thus other factors may be responsible for the faster mIPSCs in MSNs from mice with β3 subunit conditional deletion.

## DISCUSSION

Synaptic, perisynaptic, and extrasynaptic GABA_A_ receptors have distinct roles in mediating key aspects of neuronal microcircuitry that have been extensively characterized in the cerebellum (Farrant and Nusser, 2005) and the thalamus (Jia et al., 2007). These roles include mediating fast feedforward and feedback inhibition, dendritic shunting, regulation of excitability (Semyanov et al., 2004), and generation of oscillatory activity (Cobb et al., 1995; Leresche et al., 2012). Striatal MSNs have crucial roles in controlling movement and are implicated in several neurological disorders (Graybiel et al., 1994). GABA is critical to striatal function as MSNs are GABAergic in nature, forming inhibitory collateral axons among themselves (Gittis and Kreitzer, 2012). Further, GABAergic interneurons make extensive synaptic connections within the entire nucleus. Yet the role of GABAergic inhibition in this crucial area is poorly understood. Our results extend recent important findings on the dynamics of GABAergic inhibition and GABA_A_ receptors in striatal neurons.

The power of mouse genetics has allowed in recent years the ability to identify distinct subtypes of GABAergic interneurons by expressing fluorescent markers (Tepper et al., 2010; Gittis and Kreitzer, 2012). We recently reported that NGF-NPY interneurons and the FS-PV interneurons have high synaptic connectivity with MSNs and exhibit nicotinic acetylcholine receptor mediated responses (Luo et al., 2013). In the present study, we refined those results and quantified the differences in both rise and decay time of evoked IPSCs in MSNs for presynaptic NGF-NPY and FS-PV interneurons. In Luo et al. (2013), we have also shown that low frequency (<5 Hz) activation of NGF-NPY can produce sustained GABAergic currents in MSNs. This is different from FS-PV interneurons that require higher presynaptic firing rate. We confirmed and better quantified those results in the present study. We also show that higher presynaptic firing of NGF-NPY interneuron will produce massive GABAergic conductance. However one should consider that our studies were performed at room temperature, which may differently regulate GABA transporter and thus synaptic and extrasynaptic GABA affecting the integration of IPSCs. The question that arises from these data is what GABA_A_ receptors are activated by distinct presynaptic interneurons. Development of mice that allow fluorescent identification of both interneurons and MSNs with GABA_A_ receptor subunit specific deletions are required to answer this question. Our results however, make a first contribution to this answer by showing that removal of the δ but not the β3 subunit affects the response of extrasynaptic receptor selective agonist. This is in contrast to the result that deletion of the β3 subunit but not the δ subunit affects the prolongation of synaptic decay by the general anesthetic etomidate.

Taken together these data allow us to partially model the subunit composition of synaptic and extrasynaptic GABA_A_ receptor in striatal MSNs, and propose that synaptic but not extrasynaptic GABA_A_ receptor must contain the β3 subunit. In contrast, the δ subunit is restricted to extrasynaptic GABA_A_ receptors as expected from current evidence in other brain areas. What is the role of δ and β3 subunits in perisynaptic receptors? Current models propose perisynaptic receptors as central mediators to the synaptic action of neurogliaform neurons in the hippocampus (Capogna and Pearce, 2011). Clearly more studies are needed to extend these findings to other brain regions. The data presented here, suggests that removal of the β3 subunit affect the synaptic action of neurogliaform neuron similarly in the striatum, by significantly reducing the occurrence of spontaneous slow IPSCs (Fisher’s Exact Test). The infrequent finding of MSNs with slow sIPSCs suggests that the faster decay of synaptic currents associated with β3 subunit deletion in MSNs is not related to diminished activity at NPY-NGF and MSN synapses, but rather to enhanced expression of α1 subunit-containing synaptic receptors as we previously proposed (Janssen et al., 2011). The data presented show that this effect is indeed specifically due to the deletion of the β3 subunit. In summary, this work emphasizes that in order to better understand GABAergic control of striatal microcircuitry, we need to consider the activation mode and heterogeneity of presynaptic interneurons together with the presence of specific GABA_A_ receptor subtypes with distinct function.

## Conflict of Interest Statement

The authors declare that the research was conducted in the absence of any commercial or financial relationships that could be construed as a potential conflict of interest.

## References

[B1] Ade K. K. Janssen M. J. Ortinski P. I. Vicini S. (2008). Differential tonic GABA conductances in striatal medium spiny neurons. *J. Neurosci.* 28 1185–1197 10.1523/JNEUROSCI.3908-07.2008 18234896 PMC6671393

[B2] Brickley S. Cull-Candy S. Farrant M. (1996). Development of a tonic form of synaptic inhibition in rat cerebellar granule cells resulting from persistent activation of GABA_A_ receptors. *J. Physiol. (Lond.)* 497 753–759 9003560 10.1113/jphysiol.1996.sp021806 PMC1160971

[B3] Brown N. Kerby J. Bonnert T. P. Whiting P. J. Wafford K. A. (2002). Pharmacological characterization of a novel cell line expressing human α4β 3 GABA_A_ receptors. *Br. J. Pharmacol.* 136 965–974 10.1038/sj.bjp.0704795 12145096 PMC1573424

[B4] Capogna M. Pearce R. A. (2011). GABA A, slow: causes and consequences. *Trends Neurosci.* 34 101–112 10.1016/j.tins.2010.10.005 21145601

[B5] Cobb S. R. Buhl E. H. Halasy K. Paulsen O. Somogyi P. (1995). Synchronization of neuronal activity in hippocampus by individual GABAergic interneurons. *Nature* 378 75–78 10.1038/378075a0 7477292

[B6] Cui G. Jun S. B. Jin X. Pham M. D. Vogel S. S. Lovinger D. M. (2013). Concurrent activation of striatal direct and indirect pathways during action initiation. *Nature* 494 238–242 10.1038/nature11846 23354054 PMC4039389

[B7] English D. F. Ibáñez-Sandoval O. Stark E. Tecuapetla F. Buzsáki G. Deisseroth K. (2011). GABAergic circuits mediate the reinforcement-related signals of striatal cholinergic interneurons. *Nat. Neurosci.* 15 123–130 10.1038/nn.2984 22158514 PMC3245803

[B8] Farrant M. Nusser Z. (2005). Variations on an inhibitory theme: phasic and tonic activation of GABAA receptors. *Nat. Rev. Neurosci.* 6 215–229 10.1038/nrn1625 15738957

[B9] Ferguson C. Hardy S. L. Werner D. F. Hileman S. M. Delorey T. M. Homanics G. E. (2007). New insight into the role of the beta3 subunit of the GABAA-R in development, behavior, body weight regulation, and anesthesia revealed by conditional gene knockout. *BMC Neurosci. *8 85 . 10.1186/1471-2202-8-85 PMC2100059 17927825

[B10] Forcelli P. A. Janssen M. J. Vicini S Gale K. (2012). Neonatal exposure to antiepileptic drugs disrupts striatal synaptic development. *Ann. Neurol.* 72 363–372 10.1002/ana.23600 22581672 PMC3421036

[B11] Gittis A. H. Kreitzer A. C. (2012). Striatal microcircuitry and movement disorders. *Trends Neurosci.* 35 557–564 10.1016/j.tins.2012.06.008 22858522 PMC3432144

[B12] Glykys J. Mody I. (2007). Activation of GABA_A_ receptors: views from outside the synaptic cleft. *Neuron* 56 763–770 10.1016/j.neuron.2007.11.002 18054854

[B13] Gong S. Doughty M. Harbaugh C. R. Cummins A. Hatten M. E. Heintz N. (2007). Targeting Cre recombinase to specific neuron populations with bacterial artificial chromosome constructs. *J. Neurosci.* 2 9817–9823 10.1523/JNEUROSCI.2707-07.2007 17855595 PMC6672645

[B14] Graybiel A. M. Aosaki T. Flaherty A. W. Kimura M. (1994). The basal ganglia and adaptive motor control. *Science* 265 1826–1831 10.1126/science.8091209 8091209

[B15] Hentschke H. Benkwitz C. Banks M. I. Perkins M. G. Homanics G. E. Pearce R. A. (2009). Altered GABAA,slow inhibition and network oscillations in mice lacking the GABAA receptor beta3 subunit. *J. Neurophysiol.* 102 3643–3655 10.1152/jn.00651.2009 19846622 PMC2804419

[B16] Hevers W Lüddens H. (2002). Pharmacological heterogeneity of gamma-aminobutyric acid receptors during development suggests distinct classes of rat cerebellar granule cells in situ. *Neuropharmacology* 42 34–47 10.1016/S0028-3908(01)00158-7 11750914

[B17] Ibáñez-Sandoval O. Tecuapetla F. Unal B. Shah F. Koós, T. Tepper J. M. (2011). A novel functionally distinct subtype of striatal neuropeptide Y interneuron. *J. Neurosci.* 31 16757–16769 10.1523/JNEUROSCI.2628-11.2011 22090502 PMC3236391

[B18] Janssen M. J. Ade K. K. Fu Z. Vicini S. (2009). Dopamine modulation of GABA tonic conductance in striatal output neurons. *J. Neurosci.* 29 5116–5126 10.1523/JNEUROSCI.4737-08.2009 19386907 PMC2707274

[B19] Janssen M. J. Yasuda R. P. Vicini S. (2011). GABA_A_ receptor β3 subunit expression regulates tonic current in developing striatopallidal medium spiny neurons. *Front. Cell. Neurosci.* 5 15 . 10.3389/fncel.2011.00015 PMC3147169 21847370

[B20] Jia F. Pignataro L. Harrison N. L. (2007). GABA_A_ receptors in the thalamus: alpha4 subunit expression and alcohol sensitivity. *Alcohol* 41 177–185 10.1016/j.alcohol.2007.03.010 17521848

[B21] Kravitz A. V. Freeze B. S. Parker P. R. Kay K. Thwin M. T. Deisseroth K. (2010). Regulation of parkinsonian motor behaviours by optogenetic control of basal ganglia circuitry. *Nature* 466 622–626 10.1038/nature09159 20613723 PMC3552484

[B22] Leresche N. Lambert R. C. Errington A. C. Crunelli V. (2012). From sleep spindles of natural sleep to spike and wave discharges of typical absence seizures: is the hypothesis still valid? Pflugers Arch. 463 201–212 10.1007/s00424-011-1009-3 21861061 PMC3256322

[B23] Luo R. Janssen M. J. Partridge J. G. Vicini S. (2013). Direct and GABA-mediated indirect effects of nicotinic ACh receptor agonists on striatal neurones. *J. Physiol.* 591 203–217 10.1113/jphysiol.2012.241786 23045343 PMC3630781

[B24] Madisen L. Zwingman T. A. Sunkin S. M. Oh S. W. Zariwala H. A. Gu H. (2010). A robust and high-throughput Cre reporting and characterization system for the whole mouse brain. *Nat. Neurosci.* 13 133–140 10.1038/nn.2467 20023653 PMC2840225

[B25] Mihalek R. M. Banerjee P.K. Korpi E. R. Quinlan J. J. Firestone L. L. Mi Z. P. (1999). Attenuated sensitivity to neuroactive steroids in gamma aminobutyrate type A receptor delta subunit knockout mice. *Proc. Natl. Acad. Sci. U.S.A.* 96 12905–12910 10.1073/pnas.96.22.12905 10536021 PMC23157

[B26] Murase K. Ryu P. D. Randic M. (1989). Excitatory and inhibitory amino acids and peptide-induced responses in acutely isolated rat spinal dorsal horn neurons. *Neurosci. Lett.* 103 56–63 10.1016/0304-3940(89)90485-0 2476693

[B27] Paille V. Fino E. Du K. Morera-Herreras T. Perez S. Kotaleski J. H. (2013). GABAergic circuits control spike-timing-dependent plasticity. *J. Neurosci.* 33 9353–9363 10.1523/JNEUROSCI.5796-12.2013 23719804 PMC6618570

[B28] Santhakumar V. Jones R. T. Mody I. (2010). Developmental regulation and neuroprotective effects of striatal tonic GABA(A) currents. *Neuroscience* 167 644–655 10.1016/j.neuroscience.2010.02.048 20206233 PMC2907073

[B29] Semyanov A. Walker M. C. Kullmann D. M. Silver R. A. (2004). Tonically active GABA_A_ receptors: modulating gain and maintaining the tone. *Trends Neurosci.* 27 262–269 10.1016/j.tins.2004.03.005 15111008

[B30] Tepper J. M. Tecuapetla F. Koós, T Ibáñez-Sandoval O. (2010). Heterogeneity and diversity of striatal GABAergic interneurons. *Front. Neuroanat. * 4 150 . 10.3389/fnana.2010.00150 PMC3016690 21228905

[B31] Walker M. C. Semyanov A. (2008). Regulation of excitability by extrasynaptic GABAA receptors. *Results Probl. Cell Differ.* 44 29–48 10.1007/400_2007_030 17671772

[B32] Wlodarczyk A. I. Sylantyev S. Herd M. B. Kersanté, F., Lambert J. J. Rusakov D. A. (2013). GABA-independent GABAA receptor openings maintain tonic currents. *J. Neurosci.* 33 3905–3914 10.1523/JNEUROSCI.4193-12.2013 23447601 PMC3591781

